# A computational exploration into the structure, antioxidant capacity, toxicity and drug-like activity of the anthocyanidin “Petunidin”

**DOI:** 10.1016/j.heliyon.2019.e02115

**Published:** 2019-07-20

**Authors:** Vijisha K. Rajan, C. Ragi, K. Muraleedharan

**Affiliations:** Department of Chemistry, University of Calicut, Malappuram, 673635, India

**Keywords:** Computational biology, Natural product chemistry, Pharmaceutical chemistry, Theoretical chemistry, Computational chemistry, Food chemistry, Pharmaceutical science, Petunidin, Antioxidant, Drug score, Bond dissociation energy, Toxicity

## Abstract

A computational investigation on the structure and antioxidant property of a natural food colorant Petunidin (PT) was performed under DFT/B3LYP/6-31+ G (d, p). PT has a drug score of +0.804 which indicates its drug-like nature. The antioxidant property of PT was well explained by HAT mechanism and it has been found that the electron releasing substituents decreases the BDE value. PT has lowest BDE value at C3 position and is confirmed by the lowest pKa value, high atomic charge and lowest bond order. PT easily donates the hydrogen atom and exists in the deprotonated form in blood as the pKa value at C3 is less than the pH value of blood. PT shows no violation to Lipinski's rule of 5 indicating its nature as an orally admissible drug. More over PT has considerable bioactivity against nuclear receptor ligand while it shows only moderate activity towards GPCR and ion channel modulator. Also it shows moderate activity as an enzyme inhibitor and protease inhibitor but shows considerable activity as a kinase inhibitor. PT is non toxic in nature and all these factors favor its use as a potential antioxidant and a drug.

## 1 Introduction

The imbalance produced by the so called Reactive Oxygen Species (ROS) leads to Oxidative Stress (OS) in biological system. The ROS, including peroxy, superoxy, alkoxy and hydroxyl radicals, hydrogen peroxide and organic peroxides, are highly reactive free radicals causing damages to DNA and proteins, resulting in a number of diseases. In order to neutralize these ROS, our body has normal defense mechanisms provided by the secondary metabolites called antioxidants. Sufficient amount of antioxidants in living systems, maintain normal cellular activities, good health to heart, normal blood pressure, etc. [1, 2, 3, 4, 5, 6, 7]. The electron transfer to molecular oxygen takes place at the respiratory chain and the electron transport chain is located on the mitochondria implying that the ROS are mostly produced in mitochondria. Thus chemists and pharmaceutical experts are actively involved in research to discover prominent candidates for removing the ROS in mitochondria, i.e., to act on mitochondrial membranes.

Antioxidants are widely distributed in various parts of plants like fruits, leaves, flowers, etc., and a number of antioxidants are present in cow milk and honey [2, 8, 9]. One must include these in diet to maintain the optimum level of antioxidants in the body. Studies have shown that polyphenols (both natural and synthetic) are promising antioxidants [10, 11]. But synthetic antioxidants have lots of side effects and thus, we are focused on the natural antioxidants. Polyphenols contains hydroxyl groups which make them able to scavenge free radicals and the main mechanism for radical scavenging reaction has been found to be hydrogen atom transfer (HAT) [12].

The title compound, Petunidin (PT), is an important anthocyanidin found in plants. Anthocyanidins, a class of natural polyphenols, are major constituents of vascular plants and are harmless water soluble natural color pigments [13]. The attractive color makes their wide acceptance as good natural food colorants. They also found to have the capacity to chelate metal ions in biological systems [14] and thus they can prevent the peroxidations catalyzed by metal ions like Cu. Due to the wide range of uses, there are a number of research articles available in literature about anthocyanidins. Studies have shown that they have antioxidant, anticancer and antidiabetic capacities [15]. The advantage of using anthocyanidins as food colorants is, besides their coloring ability, they also serve as radical scavengers [16]. Another important property of anthocyanidins is that they can be used as pH indicators [17] as they show different colors at different pH values.

**Fig. 1:**
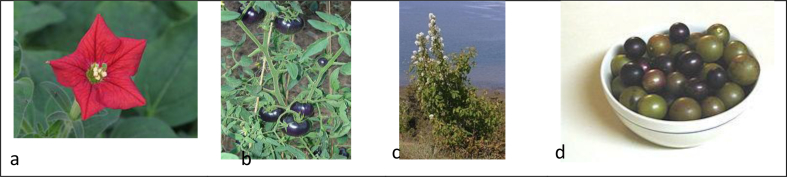
Plants containing Petunidin: (a) *Petunia exserta*, (b) BlueTomato and (c) *Amelanchier alnifolia* (d) Muscadine grapes.

Bilberries and blueberries contain high concentrations of PT and are an extremely good source of PT. Berries and grapes are widely used in wine making; for example, muscadine is the major source of PT used in making artisan wine in Florida. Moreover, some other plant based food also contains good levels of PT. Table 1 outlines some of the best food sources of PT and also reveals the presence of PT imparting color to the food materials. The name Petunidin is derived from ‘*Petunia*’, a plant containing PT (Fig. 1). When exposed to sunlight, the ‘Indigo Rose’ tomatoes become deep purple in color because of the presence of PT as the colour pigment [18]. PT is a powerful antioxidant and has potential to fight against cancer cells and also it reduces the risk of heart attack [19, 20, 21, 22, 23, 24, 25]. In order to explain these properties in a satisfactory manner, a detailed mechanistic (radical scavenging mechanism) analysis on PT is needed so that we hereby make an attempt to give a theoretical exploration into the antioxidant property of PT.

**Table 1:** Sources of PT.

Food	Mg of PT per 100g
Black beans	9.57
Bilberries	51.01
Blackcurrants	3.87
Blueberries	26.42
Cowpeas	27.82
Red grapes	2.11
Red wine	0.93

## 2 Materials and method

### 2.1 Materials

The present work had used a computational approach to study the antioxidant property of Petunidin (PT). The input structures were drawn by using the Gaussview-5.0 graphical user interface and was subjected to computational calculations in Gaussian 09 software package [26].

### 2.2 Computational methodology

Mathematical description of chemistry, called theoretical chemistry can be either computational or non-computational. In computational theoretical chemistry, the chemical problems were investigated on a computer by using suitable softwares and algorithms. There were several strategies to perform the computational works. Density Functional Theory (DFT) is one of the major and most often used computational methods to solve the chemical problems. DFT in the Kohn–Sham version is an extended Hartree Fock (HF) theory, where the many-body effect of electron correlation is modeled by a function of the electron density. DFT, which is analogous to HF, is an independent-particle model, and is comparable to HF computationally, but provides significantly better results. The main disadvantage of DFT is that there is no systematic approach to improving the results towards the exact solution.

DFT is very convenient for computational analysis because of its large accuracy and high predicting power of physical and chemical properties [16]. The level of theory adopted was B3LYP, which consists of Becke's exchange functional [27] in conjunction with Lee-Yang –Parr correlational functional [28] and the basis set used is 6–31+ G (d, p). We had done the computations both in gas as well as in solution phase. The solution phase reactions were usually carried by using the continuum models. In the Polarization Continuum Models (PCM) the solvent was described by a dielectric medium and a cavity is defined inside this dielectric medium [29]. There were different PCM models available which are designed to improve the computational performance of the method and the present work had used the IEF-PCM modelb [30].

The quantum mechanical calculations have been iteratively solved by the programs as per the keywords and the properties can be computed. These are green because it does not require any chemicals and is less time consuming. Almost all the properties can be calculated computationally with high degree of accuracy. Once the computers and softwares are built up there are no other requirements and consequently is economic too. The same set up can be used for different types of property calculations. These are the merits of computational methods over experimental tools.

The present work focused on the computational evaluation on the structure of PT and its effect on the radical scavenging property. All the computational calculations are carried out through DFT-B3LYP/6-31+G (d, p). The detailed analysis clearly explains the radical scavenging activity by means of suitable mechanisms and the color of PT was explained through Time Dependent DFT (TDDFT) analysis. Toxicological and pharmaceutical properties of PT have been carried out through the online softwares OSIRIS and Molinspiration. The following theoretical methods have been implemented for this study.

#### 2.2.1 Frontier molecular orbital (FMO) analysis

The Frontier Molecular Orbitals, including the Highest Occupied MO (HOMO) and the Lowest Unoccupied MO (LUMO) of the optimized PT have been obtained from the output file and were analyzed and the energy gap was computed. The energy gap value is an important parameter in determining the reactivity of a molecule. As the energy gap increases, less reactive will be the molecule and vice versa. The working mechanisms of an antioxidant are derived from HOMO as, a lower HOMO represents a weak electron donor and vice versa. Moreover, the hydrogen abstraction involves electron transfer, and thus the HOMO-LUMO analysis is important [31]. In the present study, we have used the HOMO-LUMO energy values for the calculation of global reactive descriptors by Koopmans's theorem. It tells that the negative of HOMO and LUMO energy directly gives the ionization potential (IP) and electron affinity (EA) of a molecule respectively [16, 32, 33, 34, 35].

#### 2.2.2 Global descriptive parameters

The relation between the chemical reactivity of a molecule and its sensitiveness to structural perturbations and responses to the changes in external conditions can be retrieved from global descriptive parameters. The global descriptive parameters include the chemical potential, electronegativity, hardness, softness, electrophilicity index, etc, [36]. The global descriptors have a significant role when comparing the properties of different molecules. The global hardness reflects the overall stability of the system [37]. The chemical hardness fundamentally signifies the reluctance towards the deformation or polarization of the electron cloud of the atoms, ions or molecules under small perturbation encountered during chemical processes. Chemical softness is the measure of capacity of a molecule to receive electrons, more precisely it is related with the groups or atoms present in that molecule and inversely proportional to chemical hardness [38]. The chemical potential in DFT, measures the escaping tendency of an electron from equilibrium, is accounted by the first derivative of energy with respect to the number of electrons [30] and is also the negative of electronegativity, which is a measure of the tendency to attract electrons in a chemical bond [38]. The electrophilic index tells us about the strength of electrophilicity of the species. The energy values of HOMO-LUMO were used to compute the global reactive descriptors by using the Eqs. (1), (2), (3), (4), (5), (6), (7), (8), and (9) [16, 35]. The IP and EA were also computed from the energy values of neutral, anion and cation moieties of PT. So, neutral, anion and cation forms of PT were taken to single point calculation and the energy values were employed for computing the IP and EA values.

(1) $$\text{Ionisation}\;\text{Potential}\left(\text{IP}\right)\;\approx \;-{\text{E}}_{\text{HOMO}}$$

(2) $$\text{Electron}\;\text{Affinity}\left(\text{EA}\right)\;\approx \;-{\text{E}}_{\text{LUMO}}$$

where E_HOMO_ is the energy of HOMO and E_LUMO_ is the energy of LUMO

Or

(3) $$\text{Ionization}\;\text{Energy}\left(\text{IE}\right)\;=\;{\text{E}}_{\text{cation}}-{\text{E}}_{\text{neutral}}$$

(4) $$\text{Electron}\;\text{Affinity}\left(\text{EA}\right)\;=\;{\text{E}}_{\text{neutral}}-{\text{E}}_{\text{anion}}$$

(5) $$\text{Hardness}\left(\eta \right)\;\approx \;\frac{\text{IP}-\text{EA}}{2}$$

(6) $$\text{Electronegativity}\left(\text{χ}\right)\;\approx \frac{\text{IP+EA}}{2}$$

(7) $$\text{Softness}\left(\text{S}\right)\;\approx \;\frac{1}{\text{2η}}$$

(8) Chemicalpotential(μ) ≈ −X

(9) $$\text{Electrophilicity}\;\text{index}\left(\text{ω}\right)\approx \;\frac{\mu^{2}}{2\eta }$$

#### 2.2.3 Natural bond orbital (NBO) analysis

A given wave function can be optimally transformed into the localized form by NBO analysis. The input atomic orbital basis set is transformed via natural atomic orbitals (NAOs) and natural hybrid orbitals (NHOs) into natural bond orbitals (NBOs). NBO is a useful tool enabling chemists to see a clear picture of both electron orbitals and population analysis. The NBO analysis provides an efficient method for studying inter and intra molecular hydrogen bonding interactions and also extent a convenient basis for investigating charge transfer or conjugative interactions in molecular system [39, 40]. The bond orders of all the bonds in a molecule can be obtained from NBO output files [16, 35].

The NBO analysis has performed on the optimized geometry of PT by giving the appropriate keyword. The bond order values were retrieved from the wiberg bond index matrix of the output file. We can also get the populations and hybridizations of atoms in PT from the same output file, but here we have not focused on those results except the bond order values. The computed bond order values are then employed to identify the weakest –OH group in PT which in turn gives the information of most stable phenoxide radical.

#### 2.2.4 Antiradical capacity

Most of the polyphenols are potential antioxidants. Both experimental and theoretical works were reported in this area. The experimental studies could give the net BDE value of a molecule only while theoretical studies could give the BDE values of all possible sites in a molecule. This makes the comparison among available antioxidants easy and the difference in reactivities can be easily understood. A number of assays like DPPH, ORAC, etc., are also available, but, each of the assay have its own disadvantages [41]. Moreover, if we are doing the theoretical studies prior to experimental studies, we could omit the unwanted steps and we have to synthesize only the most potential drug like molecule. This increases the use of theoretical methods now a day, especially, in the area of drug designing. Thus, here also, we have carried out a computational approach to evaluate the radical scavenging activity of PT. A number of molecular descriptors are available to screen the best antiradical molecule. These are HOMO-LUMO energies, Adiabatic Ionization Potential *(*AIP), Proton Dissociation Enthalpy (PDE), BDE, Proton affinity (PA) and Electron Transfer Enthalpy (ETE). Here we have discussed 3 mechanisms which describe the antiradical reactions [16, 35, 42, 43, 44, 45, 46, 47, 48, 49, 50]:

1) HAT (*Hydrogen atom transfer*) mechanism:

(10) $$\text{ArOH }+\;{\text{X}}^{\text{•}}\to {\text{ArO}}^{\text{•}}+\text{ XH}$$

According to this mechanism, the phenolic antiradical reacts directly with a free radical which is neutralized, and a radical form of phenolic antiradical appears. A numerical parameter associated with this mechanism is BDE. The lower BDE parameter characterizes the better antiradical property.

2a) SET (*Single electron transfer*) mechanism:

(11) $$\text{ArOH }+\;{\text{X}}^{\text{•}}\to {\text{ArOH}}^{\text{•}}+\;{\text{X}}^{-}$$

Here a phenolic antiradical molecule reacts with the free radical, and a cationic radical form of the phenolic antiradical and an anionic form of the radical appear. The numerical parameter related to the SET mechanism is AIP.

2b) SET-PT (*Single-electron transfer followed by proton transfer*):

(12) $${\text{ArOH}}^{\text{•+}}\to {\text{ArO}}^{\text{•}}+\;{\text{H}}^{+}$$

This mechanism is a two-step reaction. The first step is the same as that of 2a. In the second step, the cationic radical form of the phenolic antiradical decomposes into a phenolic radical and proton. The numerical parameters related to the SET-PT mechanism are AIP for the first step and PDE for the second step.

3) SPLET (*Sequential proton loss electron transfer*):

(13) $$1.\;\text{ArOH}\to {\text{ArO}}^{-}+\;{\text{H}}^{+}$$

(14) $$2.\;{\text{ArO}}^{-}+{\text{X}}^{•}+{\text{H}}^{+}\to {\text{ArO}}^{•}+\text{ XH}$$

This mechanism is also a two-step reaction. In the first step the phenolic antiradical dissociates into an anionic form and proton, and then ions created in the first reaction react with the free radical. In this reaction, a radical form of the phenolic antiradical and a neutral molecule appear. The numerical parameter related to this mechanism is for the first reaction step: PA and for the second step: ETE.

The numerical parameters corresponding to each of the above mechanisms were computed by taking the enthalpy values of the reactive species involved in each of the chemical reactions represented by Eqs. (8), (9), (10), (11), and (12). The enthalpy values were obtained from the thermochemistry part of the out file and which in turn has been used to compute the antioxidant parameters by the following equations.

(15) $$\text{BDE }={{\text{H}}_{{\text{ArO}}^{•}}}^{+}{\text{H}}_{{\text{H}}^{•}}-{\text{H}}_{\text{ArOH}}$$

(16) $$\text{AIP}={{\text{H}}_{{\text{ArOH}}^{•}}}^{+}-{\text{H}}_{\text{ArOH}}$$

(17) $$\text{PDE}={\text{H}}_{{\text{ArO}}^{•}}+{\text{H}}_{{\text{H}}^{+}}+-{{\text{H}}_{{\text{ArOH}}^{•}}}^{+}$$

(18) $$\text{PA}={{\text{H}}_{\text{ArO}}}^{-}+{\text{H}}_{{\text{H}}^{+}}-{\text{H}}_{\text{ArOH}}$$

(19) $$\text{ETE}={\text{H}}_{{\text{ArO}}^{•}}-{\text{H}}_{{\text{ArO}}^{-}}$$

After computing these values, the most suitable mechanism for explaining the radical scavenging activity of PT has been found and the effect of substituents on the radical scavenging activity has also been evaluated. These are then correlated with available experimental values.

#### 2.2.5 OSIRIS property explorer

The toxicity risks (mutagenicity, tumorogenicity, irritation and reproduction) and the physico-chemical properties such as logP, solubility (logS), molecular weight, druglikeness and drug score are calculated through OSIRIS Property Explorer online software. For screening potential drugs, the toxicological studies are highly significant and the drug score must be positive. Before going deep into the biological activities of a molecule, knowledge about its toxicity are highly beneficial [51, 52].

In OSIRIS property explorer, Drug score is calculated by summing up the scores of the individual fragments in the molecule under investigation from a list of 5300 molecular fragments. The frequency of occurrence of each fragment is decided based on a collection of 3300 drugs and 15,000 commercially available chemicals (Fluka) which are not drugs [53]. A positive drug score indicates that the molecule predominantly has fragments similar to that of drugs in use. The druglikeness, miLogP, logS, molecular weight and toxicity risks combines into a global value, called drug score, for a potential new drug candidate. It can be calculated as;

(20) $$DS=\pi \left(\frac{1}{2}+\frac{1}{2}Si\right)\pi ti$$

where

(21) $$Si={\left(1+S^{ap+b}\right)}^{-1}$$

DS is the drug score and Si is the contributions from milogP, logS, molecular weight and druglikeness (π) obtained from Eq. (20)), which is a spline curve. ‘a’ and ‘b’ are parameters for miLogP, logS, molecular weight and drug-likeness and has values (1, −5), (1, 5), (0.012, −6) and (1, 0), respectively. ‘t_i_’ is the contributions from the toxicity risk types and has values 1.0, 0.8 and 0.6 for no risk, medium risk and high risk, respectively. A positive drug score indicates that the molecule predominantly have pharmachoric groups and can be used as a potential drug.

## 3 Results and discussion

### 3.1 Optimization of structure

**Fig. 2:**
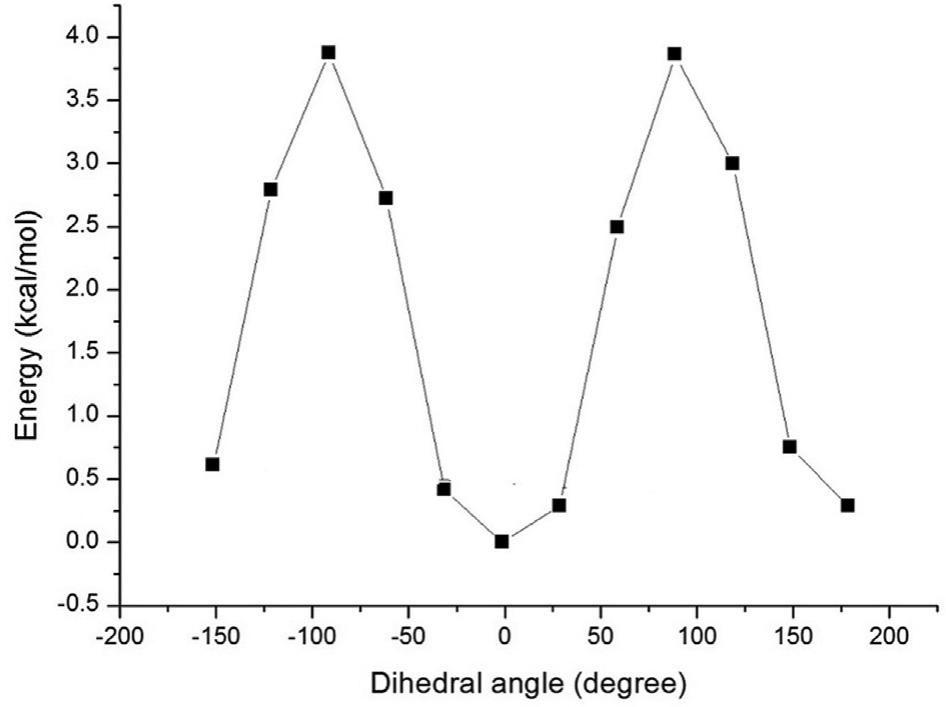
PES diagram of Petunidin.

**Fig. 3:**
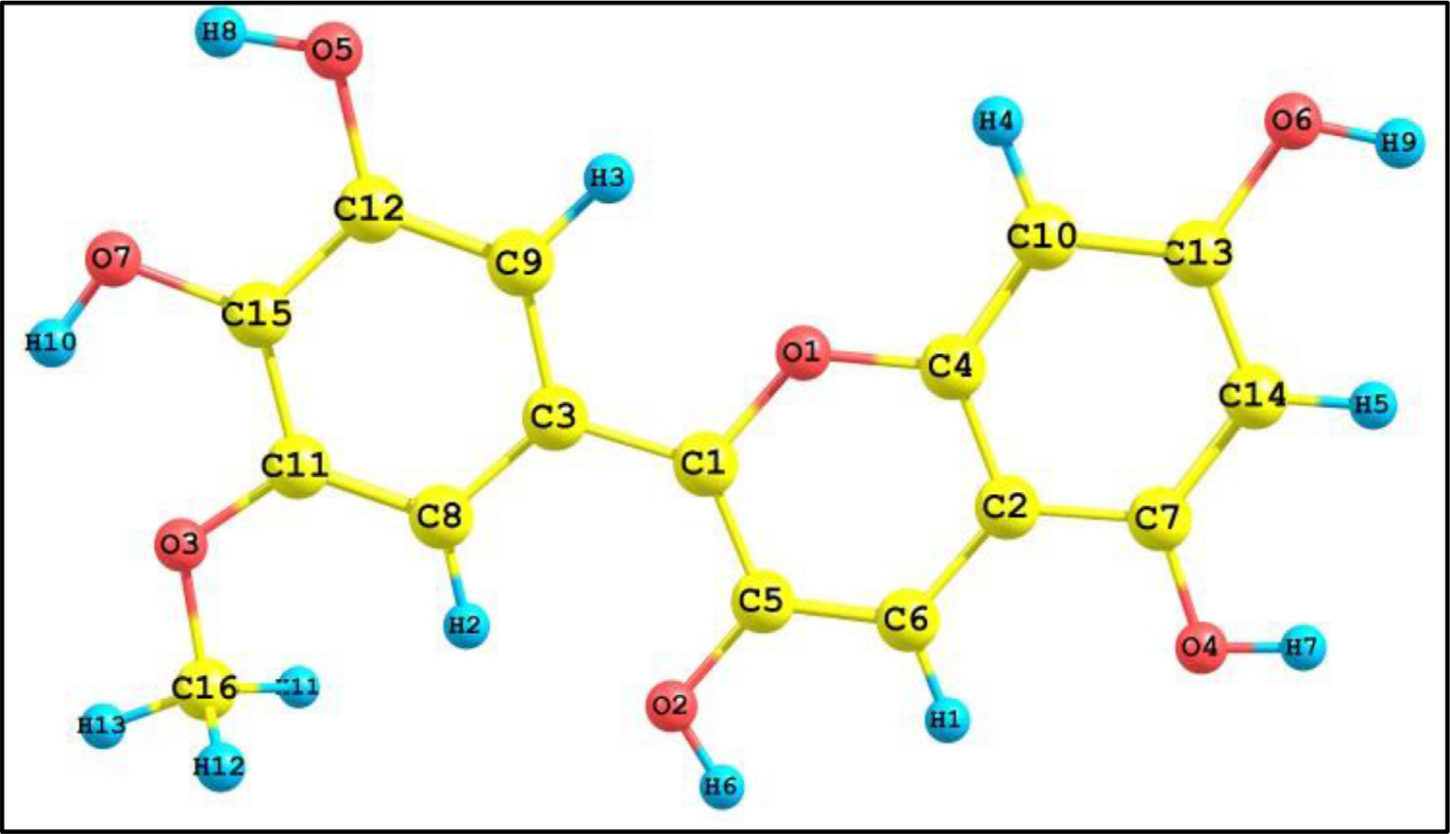
Optimized structure of stable conformer.

The basic structure of anthocyanidins consist of an aromatic ring [A] bonded to a heterocyclic ring [C] that contains an oxygen which is also bonded by a C–C bond to a third aromatic ring [B]. They belong to polyphenolic groups containing at least one –OH group in the benzene ring [16]. PT is a completely conjugated compound with a positive charge on the Oxygen atom of ring C. The planarity of PT is confirmed by the potential energy scanning of the dihedral connecting the rings B and C (Fig. 2). The lowest energy conformer is shown in Fig. 3 and the optimized geometrical parameters are given in Table 2.

**Table 2:** Geometric parameters of Petunidin.

Bond	Bond length (Å)	Bond	Bond length (Å)	Bond	Bond angle (°)	Bond	Bond angle (°)
R(1,8)	1.3497	R(10,15)	1.4238	A(8,1,11)	125.5769	A(10,15,25)	120.467
R(1,11)	1.3601	R(10,16)	1.418	A(12,2,29)	110.1304	A(18,15,25)	120.2553
R(2,12)	1.3576	R(11,17)	1.3839	A(18,3,23)	118.7638	A(10,16,19)	120.6842
R(2,29)	0.9674	R(12,13)	1.384	A(14,4,30)	111.1046	A(10,16,26)	121.3142
R(3,18)	1.3611	R(13,24)	1.0865	A(19,5,31)	109.3158	A(19,16,26)	118.0016
R(3,23)	1.4294	R(14,21)	1.3832	A(20,6,32)	111.4742	A(11,17,20)	117.2334
R(4,14)	1.3496	R(15,18)	1.3839	A(22,7,33)	108.742	A(11,17,27)	121.9403
R(4,30)	0.9675	R(15,25)	1.0766	A(1,8,10)	113.6024	A(20,17,27)	120.8262
R(5,19)	1.3508	R(16,19)	1.384	A(1,8,12)	116.9407	A(3,18,15)	126.7892
R(5,31)	0.9697	R(16,26)	1.0808	A(10,8,12)	129.4569	A(3,18,22)	112.6756
R(6,20)	1.343	R(17,20)	1.3982	A(11,9,13)	117.9594	A(15,18,22)	120.5352
R(6,32)	0.9676	R(17,27)	1.0821	A(11,9,14)	117.4908	A(5,19,16)	120.0313
R(7,22)	1.3443	R(18,22)	1.4101	A(13,9,14)	124.5498	A(5,19,22)	120.6456
R(7,33)	0.9727	R(19,22)	1.4073	A(8,10,15)	122.0384	A(16,19,22)	119.3231
R(8,10)	1.4388	R(20,21)	1.4154	A(8,10,16)	118.3301	A(6,20,17)	116.5611
R(8,12)	1.4237	R(21,28)	1.087	A(15,10,16)	119.6316	A(6,20,21)	121.9724
R(9,11)	1.4134	R(23,34)	1.0954	A(1,11,9)	118.4275	A(17,20,21)	121.4665
R(9,13)	1.4056	R(23,35)	1.0954	A(1,11,17)	117.9301	A(14,21,20)	120.184
R(9,14)	1.4263	R(23,36)	1.0892	A(9,11,17)	123.6424	A(14,21,28)	120.1139
				A(2,12,8)	118.1481	A(20,21,28)	119.7021
				A(2,12,13)	122.1817	A(7,22,18)	121.7997
				A(8,12,13)	119.6702	A(7,22,19)	117.652
				A(9,13,12)	121.4253	A(18,22,19)	120.5483
				A(9,13,24)	118.7229	A(3,23,34)	110.8421
				A(12,13,24)	119.8518	A(3,23,35)	110.8425
				A(4,14,9)	115.6234	A(3,23,36)	105.8793
				A(4,14,21)	124.3938	A(34,23,35)	109.9624
				A(9,14,21)	119.9829	A(34,23,36)	109.6156
				A(10,15,18)	119.2777	A(35,23,36)	109.6145

Before going to the mechanistic analysis of the antioxidant capacity of PT, some important structural parameters are to be discussed. The stable conformer has a dipole moment of 6.85 Debye. Dipole moment is important in studying the interaction of PT with different groups of proteins/biomolecules in plants or human body. Another important parameter is the acid dissociation constant, pKa value. This is computed by the MarvinSketch 6.2.2 application.

Table 3 shows pKa values of –OH groups at different positions in PT. This gives an idea about the H-atom donating property of a compound. The pKa value tells us how much acidic/not acidic a given hydrogen atom in a molecule is. This is important in protonation/deprotonation studies, as the knowledge of pKa value give an idea about where a given molecules is protonated and what strength of acid/base is required for that [54, 55]. More the pKa value, more the basicity and thus have high dissociation energy (DE) and vice versa. So we expect low dissociation energy for the site having low pKa value and have computed the DE of various sites and are given in the Table 3. The results are in good correlation with each other except at 4' position and is attributed to the presence of electron releasing –OCH_3_ group adjacent to it (electron density will increase the pKa value). If pH > pKa, the molecule exist in the deprotonated form and if pH < pKa, the molecule exist in the undissociated form. Here the lowest pKa value is found to be 5.99, is lower than the pH of blood so that it exist in the deprotonated form in blood and is soluble in blood also. So it can easily donate hydrogen to the free radicals in the biological system and act as a potential antiradical. This is further evaluated and explained by different computational tools and antioxidant mechanisms in the following sections.

**Table 3:** Important structural parameters of PT.

Energy	-1143.88 HF	
Dipole moment	6.85 Debye	
Position	pKa	DE (kcal/mol)
**3 –OH**	**5.99**	**390.99**
5 –OH	6.81	397.33
7 –OH	7.80	401.21
3' –OH	8.69	402.53
4' –OH	11.97	392.11

### 3.2 Frontier molecular orbital analysis

**Fig. 4:**
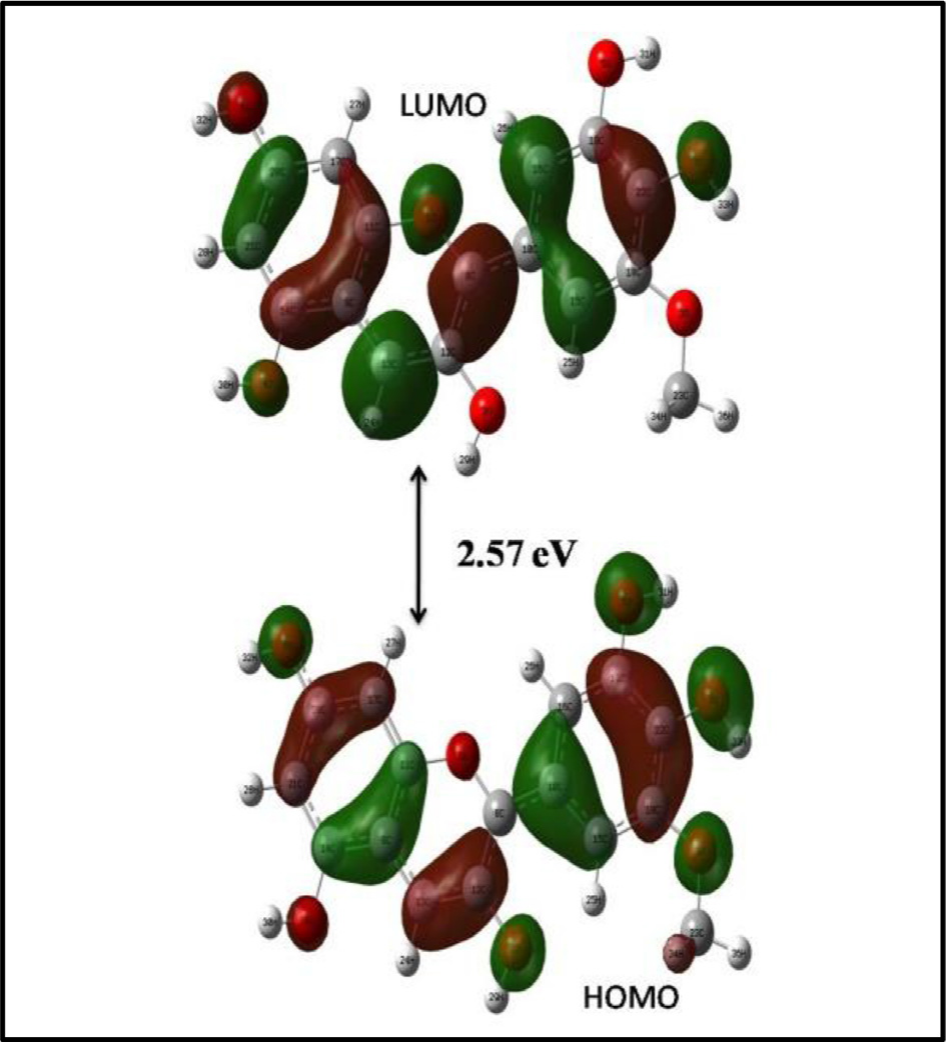
Energy gap of PT.

The molecular orbitals HOMO and LUMO are important reaction parameters as their difference gives the energy gap of the molecule. The HOMO and LUMO of PT are shown in Fig. 4. It has an energy gap of 2.57 eV which corresponds to a wavelength of 483 nm. This is responsible for the shoulder peak in its UV spectrum (given in the next section). As the antioxidant mechanisms involve electron transfer, the analysis of HOMO-LUMO is significant in identifying the active site of radical formation. A compound containing more than one –OH groups, one with highest atomic charge is the active site and which in turn is the site for the stable radical formation.

**Fig. 5:**
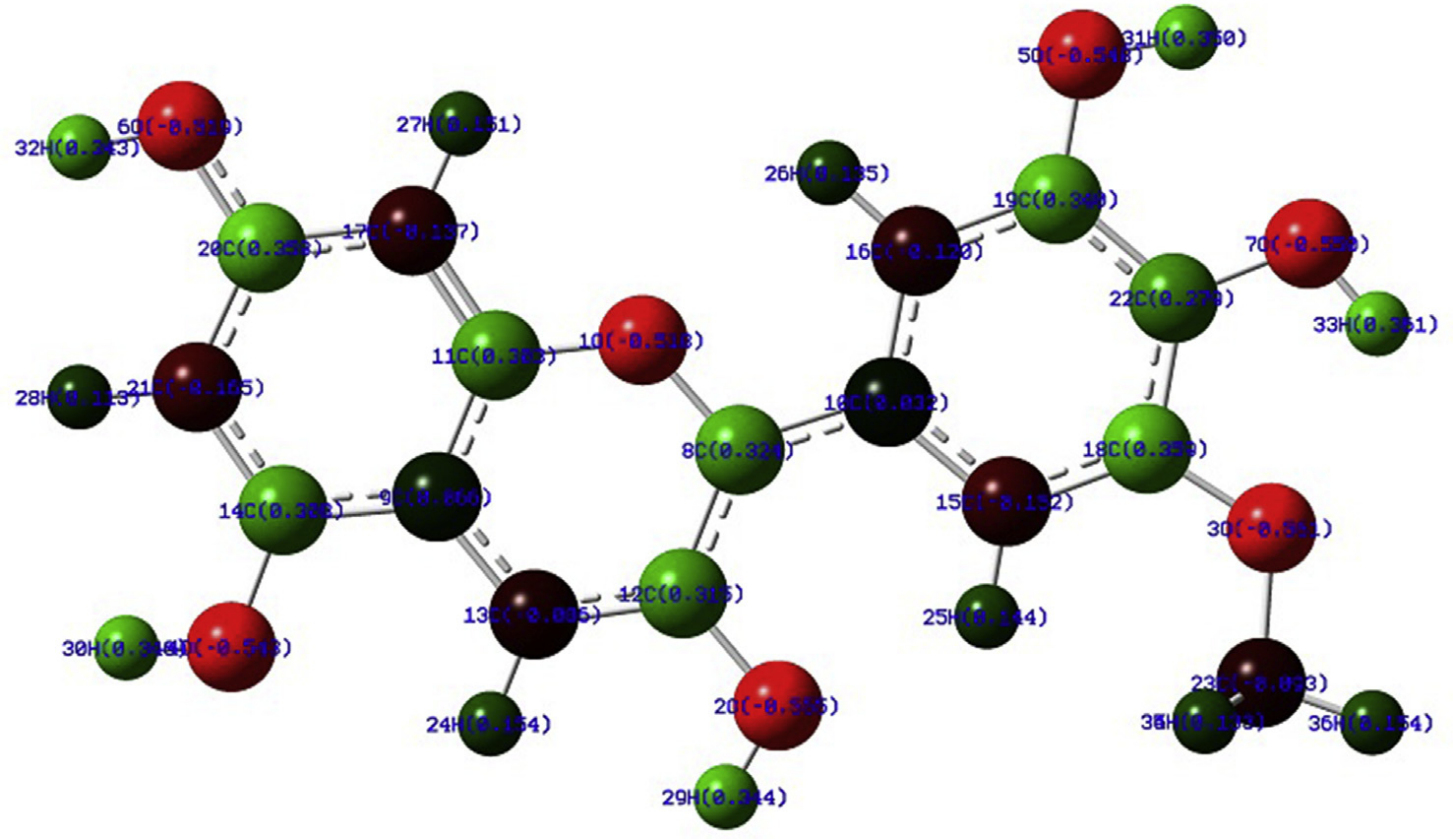
Atomic charges on PT.

The highest charge (-0.555) is on the oxygen atom of C3 position of the anthocyanidin ring so that it is the active site in PT (See Fig. 5). To confirm this, all the radicals are optimized and the lowest energetic radical or in other words the stable radical is found to form from the –OH group at C3 position. Again this is confirmed with the bond order values from NBO analysis and found that this –OH group has the minimum bond order so that it will break first to form a stable radical (see section 3.6).

### 3.3 UV-Visible spectral analysis

**Fig. 6:**
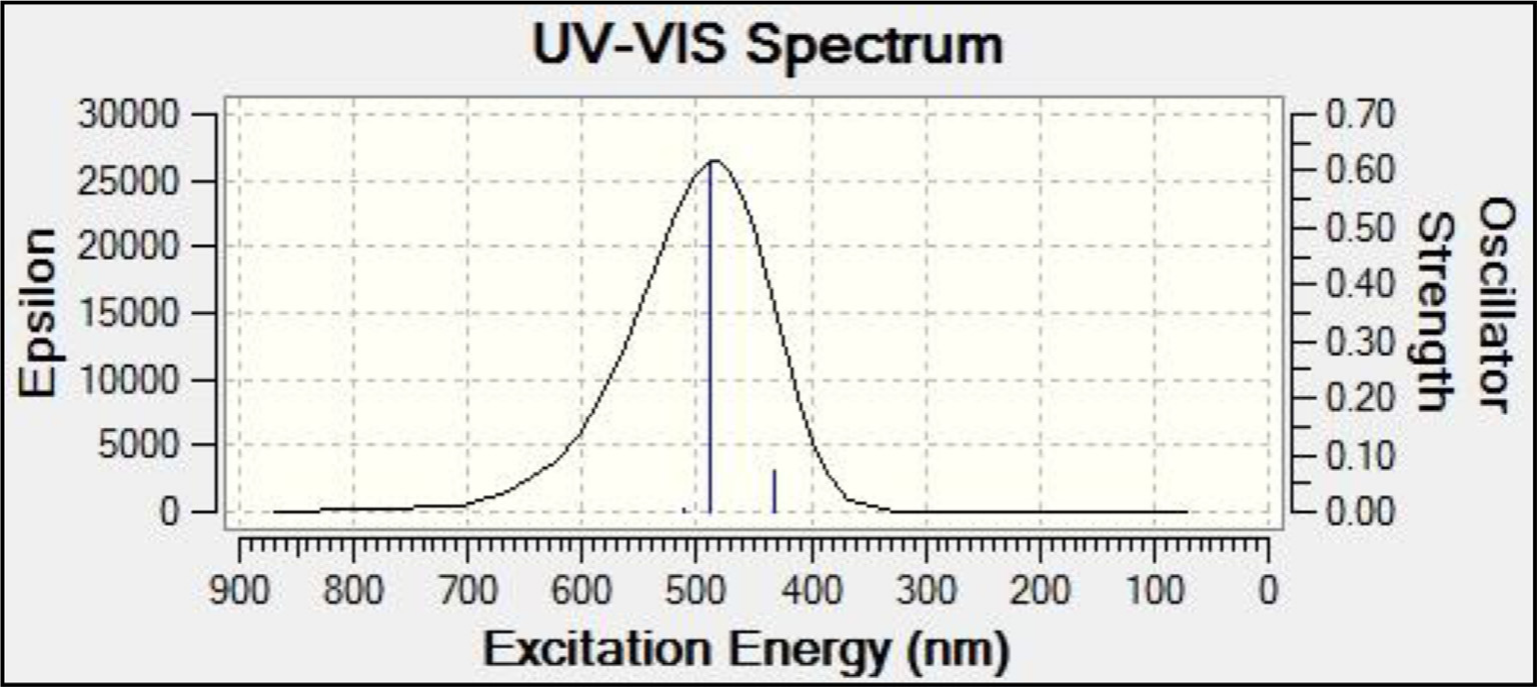
Gas phase UV-Visible spectrum of Petunidin.

The UV-Visible spectral analysis of PT has been performed by the TD DFT tool in G09 software package. The gas phase spectrum is shown in Fig. 6. It has been found that there are three peaks in the spectrum of which the λ_max_ is observed at 512 nm. This is in the blue–green region of electromagnetic spectrum so that the color of the compound will be red-purple. This is in agreement with the reddish purple color of plants containing PT. The other peaks are at 488 and 431nm.

In aqueous phase, the λ_max_ is shifted towards lower wavelength region (498.28 nm). The other peaks are 466.38 and 427.78 nm. This is because of the increase of energy gap to 2.71 eV in solution phase.

### 3.4 Global reactive descriptor analysis

The global reactive descriptors of PT are given Table 4. The energy gap of PT (2.57 eV) is smaller than that of flavonols so that PT is highly reactive. The anthocyanidin are sensitive to pH and other environmental factors. The parameters can be computed in two methods: 1) by orbital methods and 2) by energy (of molecular species involved the respective reaction i.e., anion, cation, neutral) method. The relatively small value for hardness and high value of softness makes PT reactive. For comparison, the corresponding values of Quercetin (Q), the most studied flavonoid, under same level of theory and basis set is given in Table 4.

**Table 4:** Global reactive descriptors of PT.

Descriptors	Values (eV) of PT		Values (eV) of Q	
	Orbital method	Energy method	Orbital method	Energy method
IE	8.73	9.97	5.46	6.71
EA	6.16	4.87	1.85	0.55
Η	1.29	2.55	1.81	3.08
S	0.39	0.19	0.27	0.16
Χ	7.44	7.42	3.65	3.63
Μ	-7.44	-7.42	-3.65	-3.63
Ω	29.54	10.79	3.69	2.13

The results show that PT is reactive than Q and thus PT have lower stability than Q. The positive sign in PT makes its EA values so high in comparison with Q, which indicate that PT is a good electron accepter than Q. This is reflected in their IE and χ values also. Whenever, there is a chance of undergoing charge transfer or electron transfer reactions, PT will prefer to accept electrons rather than donating them.

### 3.5 Antioxidant capacity of PT

The specialty of computational method of predicting the antioxidant properties of compounds is that we could get the BDE value of all the possible sites of the molecule which is not possible with experimental studies. In PT there are five –OH groups and can form radicals by donating hydrogen atom to the free radicals in the biological system. Numerical parameters related to all the antioxidant mechanisms are given in Table 5.

**Table 5:** Parameters of all the antioxidant mechanisms.

Parameter	Value (kcal/mol)
BDE gas	78.72
BDE water	77.02
AIP	229.91
PDE	162.56
PA	-112.68
ETE	505.15

Table 5 reveals that all the parameters except BDE are high so that those mechanisms are not suitable for explaining the radical scavenging activity of PT [56]. Thus the most suitable mechanism for explaining the radical scavenging activity of PT is HAT and the related parameter is BDE. Lower the BDE value, greater is the antioxidant capacity. BDE value is computed in both gaseous as well as in aqueous phase. Also the BDE values of all the possible sites have been computed and is given in Table 5. As the anthocyanidins are water soluble, we expect a lower BDE value in aqueous phase and are true here also. The advantage of water soluble drugs is that the excess concentration can be excreted through urine.

For PT, the lowest BDE value is observed on position 3 and it follows the order 3 < 4' < 5 < 7 < 3'. The lower BDE value at position 4' is due to the presence of electron releasing –OCH_3_ group. In order to confirm this we have replaced the –OCH_3_ group with –NH_2_, a more electron releasing group than –OCH_3_ and the BDE values are computed (Table 6). It is clear from Table 6 that the BDE values of all sites are decreased on substitution by –NH_2_ group. Again the effect of substitution with glucose units have also been performed and the corresponding BDE values are computed (Table 6). Here also the BDE values decreases on glucose substitution indicating better antioxidant capacity for PT-3-glucoside than PT. This is confirmed by the experimental work done by Marja and Marina [57], where they have carried out DPPH assay for some anthocyanidins and their glucose derivatives. They found that the antioxidant activity of PT increases on glucose substitution.

**Table 6:** BDE values of all possible sites.

Position of -OH	BDE (kcal/mol)		PT-3-glu
	With –OCH_3_	With –NH_2_	
3	78.72	74.26	-
5	85.06	80.21	82.03
7	88.94	84.18	85.92
3’	90.25	89.59	88.11
4’	79.84	79.01	78.06

### 3.6 NBO analysis

In order to get the bond order values of all the –OH groups in PT, the NBO tool in Gaussian 09 has been used. From the analysis of different mechanisms for antioxidants, the most suitable mechanism for explaining the radical scavenging activity of eriodictyol is found to be HAT. In this scenario, knowledge about the bond strengths of –OH groups in eriodictyol is particularly relevant. The atomic charge analysis shows that the most active site is position 3 and which in turn has the lowest BDE value, so that, this site is responsible for the formation of a stable radical. This is confirmed by structural optimizations of radicals, that this radical is having the lowest energy. The radical at position 3 have lowest dissociation energy (DE) and lowest pKa value. Again the strength of a bond can be found by bond order analysis also. This is carried out by NBO tool in G09 and is given in Table 7.

**Table 7:** Bond orders of all the –OH bonds in PT.

Position of –OH bond	Bond order
**3**	**0.6825**
5	0.7180
3'	0.7270
7	0.7195
4'	0.7100

Table 7 display the bond order values of the –OH groups in PT. The lowest bond order value has been observed for the –OH group at position 3 and is the weakest bond. This is in agreement with the energy values obtained in the optimization studies of radicals and BDE values. So, the most active site is the –OH group at C3 position and the radical is formed first at this position. This is in agreement with atomic charge analysis also.

### 3.7 Pharmacokinetic properties of PT

The pharmacokinetic properties of PT have been computed by using Molinspiration online software and the Lipinski rule of 5 (RO5) has been validated. According to the rule, an orally admissible drug-like molecule must have:

1. HBD < 5
2. HBA < 10
3. MW < 500 Dalton
4. LogP < 5
5. ROTB < 10 (added by Veber)

Even though this rule does not predict whether a compound is pharmacologically active, it would help to get a deep knowledge about the pharmacokinetics including Absorption, Distribution, Metabolism, and Excretion (ADME) of the molecule under investigation. By this way, researchers or pharmaceutics screen out the best orally admissible drug like candidates with good ADME characteristics [58]. PT has molecular weight less than 500 Dalton and LogP value is less than 5.

The value of total polar surface area (TPSA) is also given in Table 8. It helps to understand about the drug absorption, including intestinal absorption, bioavailability, Caco-2 permeability and blood-brain barrier penetration. For a drug like molecule, the TPSA must be less than 140 A^2^ [59]. Here the TPSA value is less than the permitted values. The number of rotatable bonds (nROTB) decides the conformational flexibility of a molecule. This is quite important for analyzing the conformational changes that can be undergone by a molecule and ultimately it is important in binding the molecules to receptors or channels. Oral viability criteria has set the number of rotatable bonds to be less than or equal to 10 [53,60,61]. The nROTB in PT is 2. PT did not show any violation to the Lipinski's rule of 5 and is thus druggable in nature.

**Table 8:** Pharmacokinetic properties of PT.

Properties	Values
miLogP	-0.73
TPSA	121.54
nAtoms	23
MW	317.27
nHBA	7
nHBD	5
nViolations	0
nROTB	2
Volume	260.36

PT is analyzed under four criteria of known successful drug activity; GPCR ligand activity, ion channel modulation, kinase inhibition activity and nuclear receptor ligand activity. Also its efficiency as a protease inhibitor and enzyme inhibitor are studied. A positive value of bioactivity score indicates considerable biological activity whereas a score between −0.5 and 0.00 indicates moderate activity and less than −0.5 is considered inactive. Table 9 clearly shows that PT has positive drug score for kinase inhibition and nuclear receptor ligand activity and so it will have considerable biological activity against these two targets. However, for all the others, the bioactivity value lies in between -0.5 and 0 which indicates moderate biological activity.

**Table 9:** Bioactivity scores against different drug targets.

Targets	Bioactivity score
GPCR ligand	-0.15
Ion channel modulator	-0.17
Kinase inhibitor	0.03
Nuclear receptor ligand	0.01
Protease inhibitor	-0.29
Enzyme inhibitor	-0.01

### 3.8 Toxicity analysis and drug score

The studies regarding the toxicity of anthocyanidins were limited and were based on the extracts obtained from fruits and vegetables. PT, being a widely accepted natural food colorant, and is stable up to a pH value of 8. In present study, we have carried out a toxicological analysis by the free online software called OSIRIS Property Explorer. The results are given in Table 10.

**Table 10:** Parameters from DataWarrior.

Property	Score
Drug score	0.804
clogP	1.980
Mutagenic	None
Tumorigenic	None
Reproductive effective	None
Irritant	None
Druglikeness	1.310
Solubility	-2.338

Table 10 clearly shows that PT is nonmutagenic, nontumorigenic and nonirritant. Solubility is important in the evaluation of drug absorption and distribution characteristics. Low solubility implies low absorption. For most of the commercially available drugs, the solubility is found to be greater than -4.00 [53]. PT shows a good solubility (of -2.328). The positive value for drug score (0.804) indicate that PT can act as a potential drug. Thus PT can be used as a potential antioxidant without any side effects to biological system. Along with the coloring property, PT in food items also serves as a potential antioxidant too.

## 4 Conclusions

The article summarizes the computational investigation on the structure and antioxidant property of an anthocyanidin, Petunidin (PT). The work was performed under DFT/B3LYP/6-31+G (d, p) in both gases as well as in aqueous phase. The toxicological analysis by OSIRIS property explorer has shown that PT is nontoxic, nonmutagenic, nontumorigenic and nonirritant. PT has a drug score of +0.804 which indicates that it has considerable drug likeness which are comparable with the existing drugs. The antioxidant property of PT can be well explained by HAT mechanism with a BDE value of 78.72 and 77.02 kcal/mol in gaseous and aqueous phase respectively. The BDE values are greatly influenced by the nature of substituents and found that the electron releasing substituents decreases the BDE value. So one can tailor the antioxidant capacity of PT. PT has lowest BDE value at C3 position and is further supported by the pKa values, atomic charge analysis and bond order analysis from NBO. The most active site (C3) have the lowest pKa value and is found to be less than the pH value of blood so that it exists in blood as deprotonated. The study is very useful as the title compound is a widely accepted natural food colorant. PT shows no violation to Lipinski's rule of 5 indicating its nature as an orally admissible drug. Moreover PT has considerable bioactivity against nuclear receptor ligand while it shows only moderate activity towards GPCR and Ion channel modulator. Also it shows moderate activity as an enzyme inhibitor and Protease inhibitor but shows considerable activity as a kinase inhibitor. All these factors favor its use as a potential antioxidant and an orally admissible drug. Further the study can be extended to molecular docking analysis with protein like Mono Amine Oxidase B and also to study the metal chelation capacity to prevent the catalysis of peroxidation reactions by metal ions.

## Declarations

### Author contribution statement

Vijisha K. Rajan, Ragi C., Muraleedharan K: Conceived and designed the experiments; Performed the experiments; Analyzed and interpreted the data; Contributed reagents, materials, analysis tools or data; Wrote the paper.

### Funding statement

Vijisha K. Rajan was supported by UGC.

### Competing interest statement

The authors declare no conflict of interest.

### Additional information

No additional information is available for this paper.
